# Durability of Prestressed Piles in a Leachate Environment

**DOI:** 10.3390/ma17112497

**Published:** 2024-05-22

**Authors:** Yu Wang, Min Deng, Rihong Zhang, Xuming Yu, Junzhong Xue, Jing Zhang

**Affiliations:** 1 College of Materials Science and Engineering, Nanjing Tech University, Nanjing 211800, China; 202262103005@njtech.edu.cn; 2 Faculty of Architectural, Civil Engineering and Environment, Ningbo University, Ningbo 315000, China; zhangrh@zcone.com.cn; 3 Ningbo ZCONE High-Tech Holdings Co. Ltd., Ningbo 315000, China; 4 CGN Solar Energy (Jiaxing) Co., Ltd., Jiaxing 314000, China; xl.xujia@163.com (X.Y.); xue_jz@126.com (J.X.); 5 SGIDI Engineering Consulting (Group) Co., Ltd., Shanghai 200093, China; zhangjing@sgidi.com

**Keywords:** prestressed piles, harsh environments, leachate, durability

## Abstract

Prestressed pipe piles are common concrete components characterized by dense concrete structures and favorable mechanical properties, and thus, extensively used as coastal soft soil foundations. However, their durability in harsh environments has not been fully clarified. In this study, leachate from an actual landfill site was collected from the east coast of China as the corrosive medium, and the corrosion process was accelerated by electrifying prestressed pipe piles. The results demonstrated that the concentration of chloride ions in the concrete of the prestressed pile increased with the increase in corrosion time. Moreover, the experimental corrosion of these prestressed piles in the drying–wetting cycle proved to be the most severe. However, a protective layer of epoxy resin coating can effectively inhibit the diffusion of chloride ions into the interior of the piles. The final theoretical corrosion amounts of the piles were 1.55 kg, 1.20 kg, and 1.64 kg under immersion, epoxy resin protection, and a drying–wetting cycle environment. The application of epoxy resin reduced chloride penetration by 22.6%, and the drying–wetting cycle increased chloride penetration by 5.8%, respectively, with corresponding corrosion potentials following similar patterns. The actual corrosion depth of the welding seam was 3.20 mm, and there was a large corrosion allowance compared with the requirement (6.53 mm) for the ultimate bending moment. In summary, these prestressed piles exhibited good durability in a leachate environment.

## 1. Introduction

With the acceleration of urbanization in China, the demand for land resources has increased dramatically, and an increasing number of buildings have to be constructed in harsh landfill environments [1,2]. As a common category of foundations, prestressed concrete pipe piles are inevitably used in landfills. In landfills, domestic and industrial wastes can form leachate under the combined action of rainwater and surface runoff. The leachate accumulates in the soil layer of landfills and is not easily absorbed. Further, the underground soil of landfills fills with leachate in the long run. Landfill leachate constitutes a weak alkaline solution with a complex composition and many hazardous ions, and the corrosive media include Cl^−^, SO_4_^2−^, NH_4_^+^, and organic biomass [3]. Due to the inherent pores and microcracks in the concrete, these corrosive media may penetrate the interior of reinforced concrete materials, which would affect the durability of the concrete, thus reducing the service life of concrete structures [4,5,6].

Compared with ordinary concrete structures, prestressed concrete piles require a series of special processes, such as prestressing [7], centrifugation [8,9], and steam curing [10,11,12]. They have a higher density, lower porosity, and greater bending capacity [13,14]. Special production processes and hollow shapes contribute to the superiority of these piles over ordinary reinforced concrete in terms of microstructures and mechanical properties. In particular, prestressed high-performance concrete (PHC) piles are generally considered to have favorable durability.

In recent years, scholars have conducted in-depth research on the durability of prestressed concrete piles. In 1965 and 1970, a Japanese company constructed two factory buildings, A and B, using prestressed concrete (PC) piles as pile foundations. The durability of relevant units in the factory buildings and the PC piles in the foundation was investigated 32–37 years after operation [15]. It was found that the compressive strength of the concrete of these piles was still higher than the design requirement. The elastic modulus was higher than 4 × 10^4^ MPa, the maximum neutralization depth was 1 mm, and the tensile strength of the steel reinforcement inside the pile was not significantly reduced. Japan’s Kao Group conducted a field exposure test on the long-term durability of PC piles in 1968 [16,17,18]. The pile concrete was mixed with calcium chloride equivalent to 0.05%, 0.5%, 1.0%, 2.0%, and 4.0% of the cement mass. After 32 years, the PC steel bar elongation of the test piles with 4.0% of calcium chloride added was less than 1%; after 50 years, the PC steel bar of the test piles with 4.0% of calcium chloride added fractured. No obvious rust was found on the PC steel bars of the rest of the test piles, and the depth of neutralization was less than 2 mm. Zhan et al. [19] investigated the effects of maintenance and admixture on the durability of prestressed piles. They found that the prefabrication of the prestressed piles with a polycarboxylic water reducer and 8 h steam curing provided the highest cost-effectiveness in terms of durability, with an electrical flux of 85 °C and a chloride ion migration coefficient of 0.54 × 10^−12^ m^2^/s. Liu et al. [20] simulated pipe pile joints immersed in 5% NaCl solution for 50 years through energized acceleration. They revealed that the seams of the joints did not crack after deterioration, and that the welding seams of the welded square pile joints rusted mildly after deterioration. Li et al. [21] predicted effects on pipe piles under a chlorine ion environment by solving the diffusion equations of the chlorine ions in the layered structure of the piles. The results confirmed that increasing the thickness of the mortar layer shortened the diffusion and corrosion periods, thereby significantly reducing the service life. Increasing the thickness of protective layers and sealing pile ends were identified as effective measures to extend the service life of prestressed concrete piles.

In underground environments, dry and wet cycles often occur, and leachate environments are no exception. In recent years, scholars have studied the effects of dry and wet cycling on concrete corrosion and concluded that dry and wet coupling cycles and the diffusion of chloride ions increased the development of cracks and pores within the concrete structure and accelerated the transport of the chloride ions through the concrete.

Epoxy resin is one of the polymer materials with the best chemical resistance, the best adhesion with all kinds of concrete, no volatile by-products from curing, small shrinkage from curing, and excellent anti-corrosion performance. Epoxy resin coating can be used for the corrosion protection of prestressed pipe piles.

In summary, prestressed piles exhibit favorable long-term durability performance in ordinary environments or mildly corrosive environments. However, there is a lack of research on the deterioration process of prestressed piles in harsh environments, such as the waste leachate field. The issue of the durability of precast piles in leachate environments is closely related to the longevity of buildings in leachate environments, and the present study is undoubtedly pioneering as leachate environments are unique environments that have emerged in the course of modern urbanization. In this study, the durability of prestressed piles in the landfill leach field was evaluated by combining the energized accelerated test with the actual situation of the project. These findings may provide a theoretical basis for the protection and application of prestressed concrete piles in landfill environments.

## 2. Materials and Methods

### 2.1. Raw Materials

In this test, P-II 52.5 silicate cement produced by Onada Cement Corp (Nanjing, China) was used. The specific surface area was 366 m^2^/kg, the initial setting time was 163 min, and the final setting time was 220 min. The slag powder was a by-product of granulated blast furnace production from Ningbo Ziheng Building Materials Technology (Ningbo, China). The ratios of cement to slag powder are shown in Table 1. The coarse aggregate used was continuously graded gravel produced by the Ningbo Guoding Mining Industry (Ningbo, China), with the size ranging from 5 mm to 32 mm. The fine aggregate used was mechanism sand produced by the Ningbo Guoding Mining Industry, with a fineness modulus of 2.8. The Point-C300 high-efficiency concrete water-reducing agent produced by Kozijie New Materials (Nanjing, China) was selected as the water-reducing agent.

The diameter of the prestressed reinforcement was 10.7 mm, and the tensile strength and elongation after the fracture of the prestressed reinforcement were 1490 MPa and 8.8%. The spiral stirrup was a cold-drawn mild steel wire with a diameter of 5.0 mm, and the tensile strength and elongation after the fracture of the spiral stirrup were 620 MPa and 4.5%.

The leachate was extracted from a landfill site on the eastern coast of China and had a brown-black color and a strong, irritating odor. The pH value was 7.9, the ammonium nitrogen content was 2.55 g/L, the sulfate content was 7.43 mg/L, and the chloride content was 3.14 g/L.

### 2.2. Prestressed Piles and the Mix Ratio of Concrete

The prestressed concrete pipe was PHC 400(95) AB, using grade C80 concrete mix, and its strength was 84.1 MPa on day 28. The outer diameter of the pipe pile was 400 mm, the thickness of the protective layer was 41 mm, and seven prestressed reinforcements were uniformly arranged.

**Figure 1:**
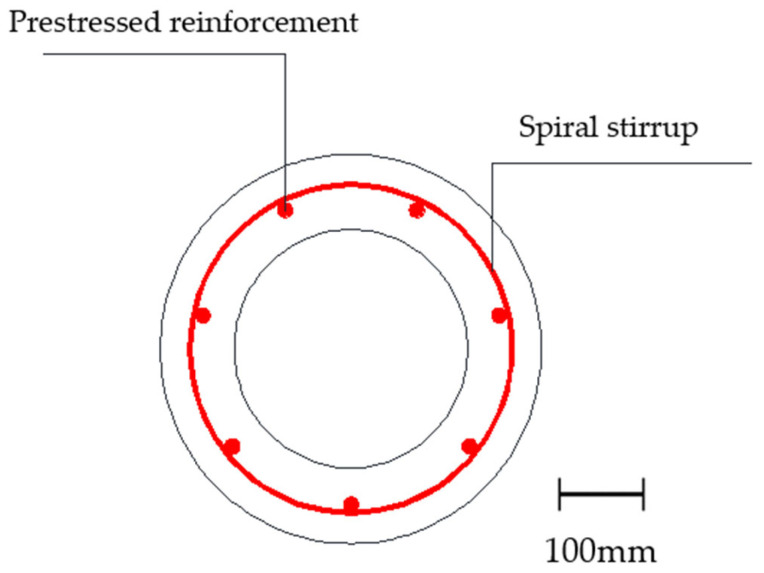
The section of prestressed pipe piles.

The concrete mixing ratio is shown in Table 2, and its quality meets the requirements of the Zhejiang Provincial Atlas G22 (*Pre-tensioned Prestressed Concrete Pipe Pile*) [22]. The reinforcement of the test pile is illustrated in Figure 1, with a pitch spacing of 80 mm for the helical reinforcement and no densified zone at either end.

**Figure 2:**
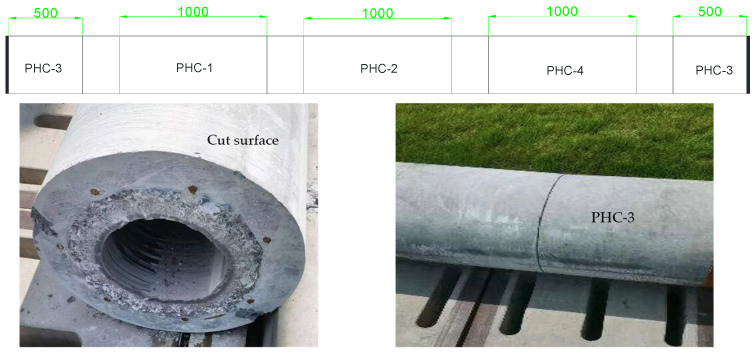
Test pile cutting diagram.

After the prestressed pile had been prepared, it underwent steam curing. Following 28 days of steam curing, the pile was cut and welded to form samples, as illustrated in Figure 2. The test piece was cut into a 1000 mm test pile.

A total of four prestressed concrete pipe piles were used in the test, named PHC-1, PHC-2, PHC-3, and PHC-4, respectively. PHC-1 was a common pipe pile with both ends blocked, PHC-2 was a pile with an epoxy coal tar protective layer applied to the body, PHC-3 was a pile formed by welding two ordinary piles together with a joint, and PHC-4 was a common pile placed in a drying–wetting cycle leachate environment with a cycle of 8 days/time. After demolding, the test pile was fabricated and cured in a steam environment (85 °C) for 6 h, with the empty holes at both ends of the piles sealed before the test.

**Figure 3:**
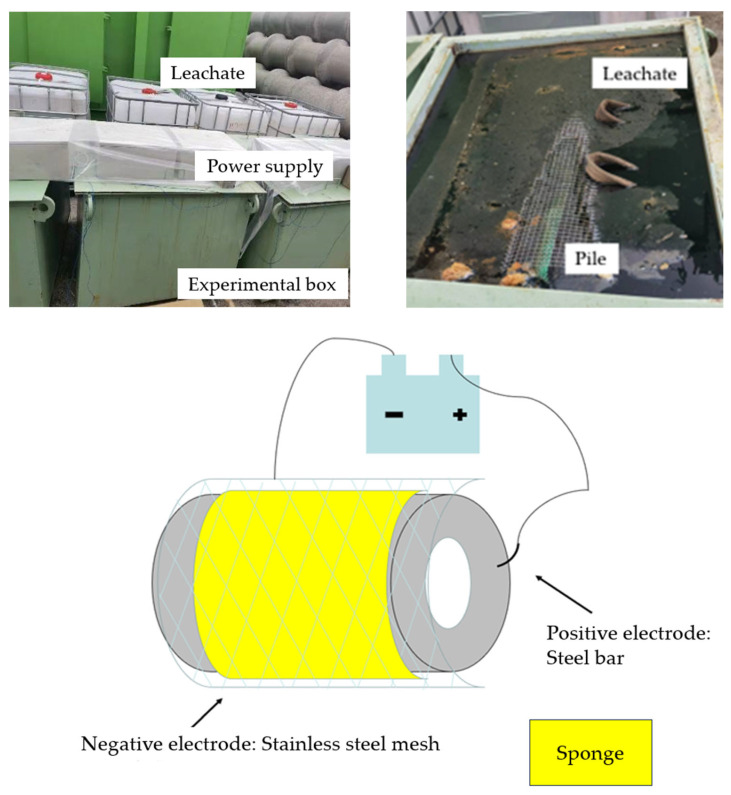
The electric acceleration device.

An electric field was applied to accelerate the migration rate of the solution ions at both ends of the pile. Electrified acceleration can increase the corrosion degree of reinforced concrete specimens in a shorter period of time compared to reinforced concrete specimens corroded for a longer time in the natural environment. Figure 3 illustrates the accelerated corrosion of a test pile, where the positive pole is the steel bar and the negative pole is the stainless steel mesh surrounding the pile.

Before the test, it was necessary to calculate the natural resistance of the piles in a harsh environment. After the test piles were placed into the maintenance box, they were submerged in the leachate from the landfill, covering the top of the test piles by at least 10 cm. After 10 days, the resistance was measured by a multimeter; the resistance results of PHC-1, PHC-2, PHC-3, and PHC-4 were 6.0 Ω, 6.2 Ω, 6.0 Ω, and 5.9 Ω, respectively. Taking 6.0 Ω as the natural resistance of the test piles in this environment, it can be calculated that with an applied voltage of 10 V, the corrosion of the piles corresponded to the corrosion for 30 years and 50 years in the natural environment after applying electricity for 96 d and 160 d.

### 2.3. Test Methods

#### 2.3.1. The Chloride Ion Concentration at Different Depths of Piles

The sampling procedure was performed with the aid of a core drilling machine. Then, the specimen was drilled to the location of the reinforcement and the pile was repaired after the coring procedure had been completed. The mortar in the concrete at the depths of 5 mm, 10 mm, 20 mm, 30 mm, and 40 mm from the surface of the test pile was ground into powder, respectively. According to JGJ/T 322-2013, *Technical specification for test of chloride ion content in concrete* [23], the mortar powder was mixed with 100 mL of distilled water, followed by stirring and boiling. Subsequently, the upper clear liquid was collected. The chloride ion content at different depths was calculated by measuring the Cl^−^ content in the clear liquid using AgNO_3_.

Samples were taken after the production of the experimental piles was complete; the concentration of chloride ions in the concrete was 0.018%. In the experiment, the chloride ion concentration was measured every 16 days. The initial chloride ion concentration of PHC-1, PHC-2, and PHC-4 was taken when they had been soaked in the leachate for 10 days without electricity, and the subsequent chloride ion concentration was measured after the accelerated corrosion of the experimental pile had been energized.

#### 2.3.2. Electric Current and Corrosion Potential

Faraday’s first and second laws describe the relationship between electrons and matter in the electrochemical process. According to the laws, the mass of the substance precipitated (or dissolved) on the electrode (Δm) is proportional to the total amount (*`*) of electricity through the electrolyte, expressed by the formula

(1) Δm=KQ

where Δ*m* is the mass of precipitated (or dissolved) material on the electrode; *K* is the electrochemical equivalent, related to the precipitated (or dissolved) substance; the electrochemical equivalent is equal to the mass of the substance precipitated (or dissolved) by 1 coulomb of electricity; and *Q* is the amount of electricity passed through the electrolyte solution.

When the total amount (*Q*) of electricity through each electrolyte is the same, the mass of each substance precipitated (or dissolved) on the electric plate (Δ*m*) is proportional to the chemical equivalent (*C*) of the substance, expressed by the formula

(2) Δm∝C

where *C* is the chemical equivalent, which is the ratio of the molar mass (*M*) of the substance to its valency (*Z*), which means that *C* = *M*/*Z*.

According to Formula (2), the following relation can be obtained by introducing Far-aday’s constant *F*:

(3) $$\Delta m=\frac{MIt}{ZF}$$

where *M* represents the molar mass of the substance (the molar mass of Fe is 56 g/mol); *I* represents the electrification current; *t* represents the electrification time; *Z* represents the valency of the substance (the valency of an iron atom is 2); and *F* represents Faraday’s constant, which has a value of 96,484 C/mol.

The product of the electric current and time can be used to calculate the mass of steel corrosion in concrete, thus assessing the corrosion of the steel in the pile. The current change was recorded three times per day in the test and the current change curve was plotted. The corrosion potential was measured using TST-XS310, a steel corrosion meter from TAster (Beijing) Testing Technology Co., Ltd. (Beijing, China), with a measuring accuracy of ±1 mV.

**Figure 4:**
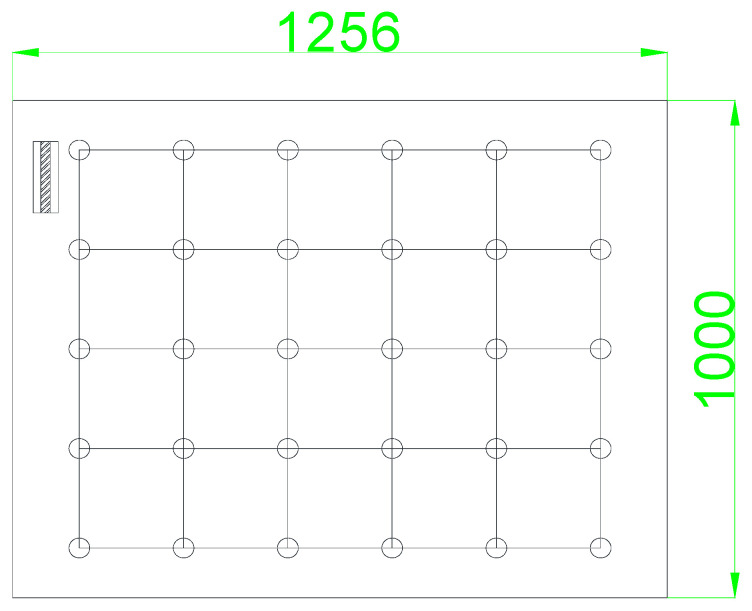
Test points of corrosion potential.

As shown in Figure 4, the test grid was first arranged on the pile surface during the corrosion potential test. The grid size was 20 × 20 cm (along the axis of the pile), with the grid intersection points serving as the test points. The DC power supply was disconnected one hour before data collection, and the wires connecting the main bar and the DC power supply were disconnected at the same time. The measured corrosion potential was compared with the corrosion potential evaluation standard to calculate the probability of steel bar corrosion.

#### 2.3.3. Changes in Weld Depths

**Figure 5:**
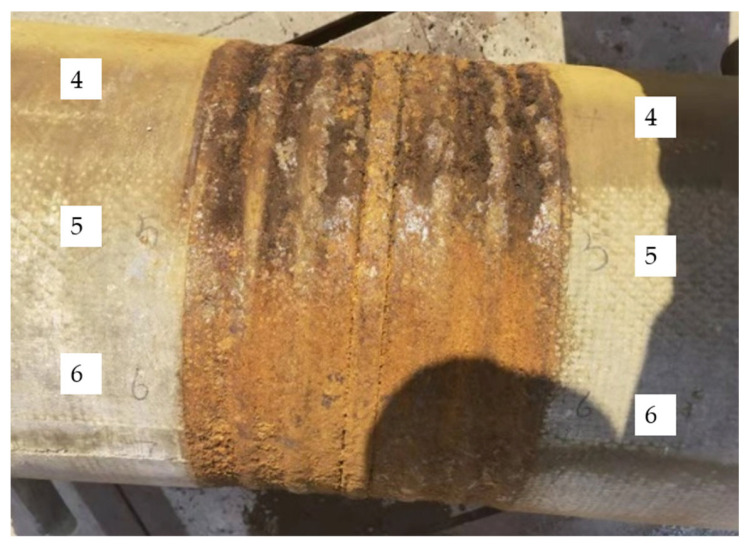
Test points at the welded joint.

Ten equidistant test points were marked at the welded joint of PHC-3. The rust pit depths were measured using a welding seam inspection ruler, and the changes in weld heights at different ages were recorded. The weld depth was measured by a weld gauge with an accuracy of 0.01 mm. Figure 5 illustrates test points 4, 5, and 6 at the welded joint.

## 3. Results

### 3.1. The Chloride Ion Concentration at Different Depths of Prestressed Piles

Chloride ions are abundant in leachate, posing significant hazards to the safety of steel bars in precast piles. The samples were taken from the depths of 0–5 mm, 5–10 mm, 10–20 mm, 20–30 mm, and 30–40 mm from the surface of the test pile in mortar.

**Figure 6:**
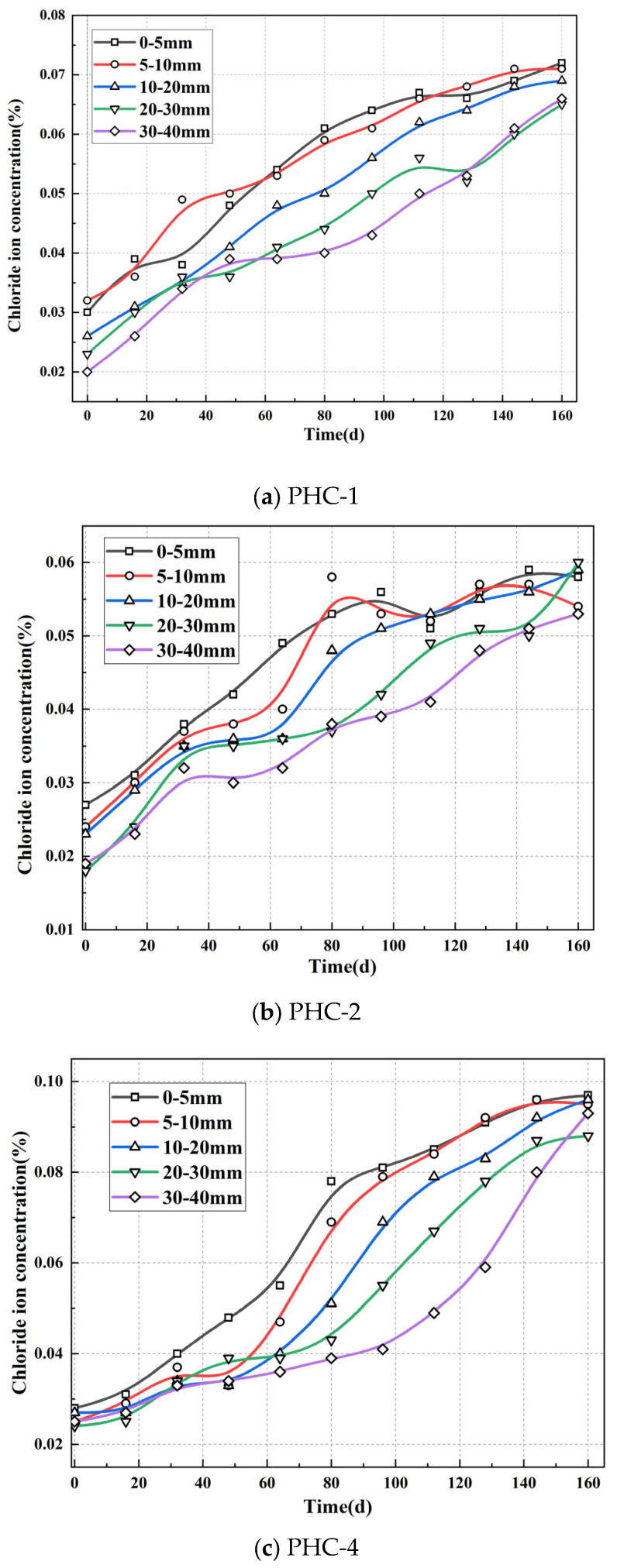
The chloride concentration at different depths of the prestressed piles.

Figure 6 illustrates the chlorine ion concentration of the prestressed piles at different depths. In the early and middle stages, the chloride ion concentration on the surface increased faster than that in the interior. In the middle and later stages, the increase rate of the chloride ion concentration accelerated in the interior, eventually reaching a uniform concentration throughout the pile.

In the early stages of corrosion testing, the chloride ion concentration of PHC-1 and PHC-2 in the leachate at a depth of 5–10 mm started to increase after 0 d, while that of PHC-4 started to increase significantly after 16 d. The chloride ion concentration inside the prestressed pile was similar to that in the surface layer in the early stage. In the middle stage, the chloride ion concentration inside the pile began to increase continuously. The chloride ion concentration inside PHC-1 and PHC-2 gradually approached the surface chloride ion concentration. The increase rate of the chloride ion concentration at the depths of 0–5 mm, 5–10 mm, and 10–20 mm in PHC-4 decelerated after 96 d. However, the chloride concentration inside the prestressed pile increased faster, and its chloride concentration at the depths of 0–5 mm and 5–10 mm increased more slowly after 160 d. The chloride concentration in the prestressed pile was found to be higher than that at the depths of 0–5 mm and 5–10 mm.

The epoxy resin protective layer inhibited leachate corrosion. From day 48, the chloride ion concentration in PHC-2 was lower than that in the other precast piles in the leachate environment. On day 0, the surface chloride ion concentration was 0.027%, while the chloride ion concentration at the depths of 5–10 mm, 10–20 mm, 20–30 mm, and 30–40 mm was 0.024%, 0.023%, 0.018%, and 0.19%, respectively, which was close to that of PHC-1 and PHC-4. On day 48, the gap between the chloride ion concentration in the surface layer of the pipe piles in PHC-2 and the rest of the leachate environment started to increase, until day 160, when the chloride ion concentration in the surface layer was only 0.059%, which was 0.010% less than PHC-1.

In terms of the chloride ion concentration, the epoxy applied to the surface of the prestressed pile inhibited 20%, 23%, 14%, 7%, and 19% of the chloride ions in the concrete 0–5 mm, 5–10 mm, 10–20 mm, 20–30 mm, and 30–40 mm from the surface, respectively, compared to PHC-1. The dry and wet cycling conditions accelerated the diffusion of the chloride ions into the concrete, increasing the chloride ions by 35%, 34%, 39%, 35%, and 41% in the concretes 0–5 mm, 5–10 mm, 10–20 mm, 20–30 mm, and 30–40 mm from the surface, respectively, compared to PHC-1.

**Figure 7:**
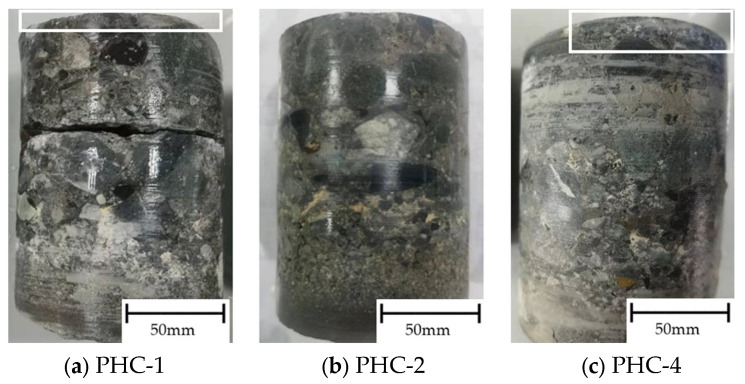
The staining of silver nitrate.

Direct titration with AgNO_3_ was performed on the core samples on day 160, as illustrated in Figure 7. The core samples of PHC-1 and PHC-4 had white precipitates at a depth of about 0–10 mm, while that of PHC-2 was protected by the epoxy resin, and the chloride ion concentration in the interior of the concrete was not enough to cause AgNO_3_ to produce white precipitates.

### 3.2. Electric Current and Corrosion Potential

The process of steel corrosion is accompanied by an increase in resistance, leading to a decrease in the current under a constant voltage. According to Faraday’s law, the amount of steel corrosion is proportional to ∑It. Therefore, the changes in the image integral caused by the current changes during the pile corrosion testing can effectively reflect the law of steel corrosion in the pile.

**Figure 8:**
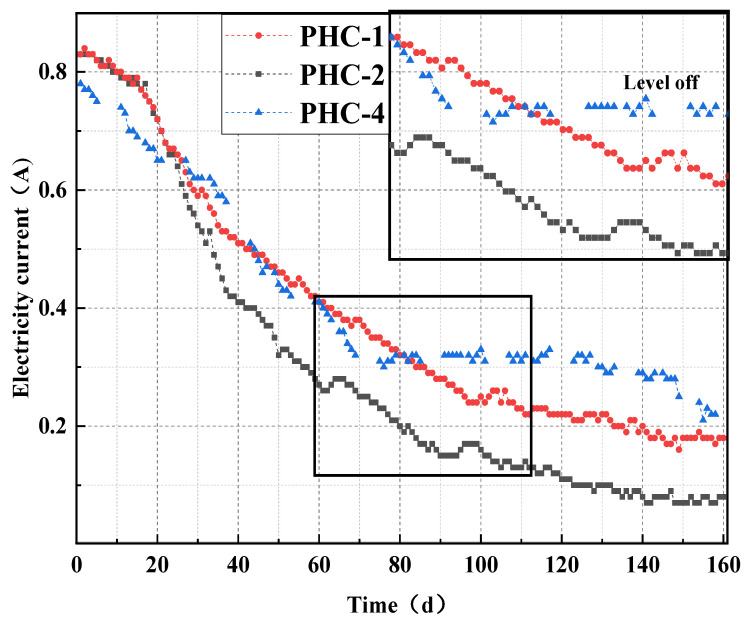
Current changes in prestressed piles.

As illustrated in Figure 8, the overall trend of the electric current in the pile foundations was decreasing and tended to level off eventually. The current integrals of PHC-1, PHC-2, and PHC-4 were 61.85 A·day, 47.45 A·day, and 65.37 A·day, respectively, from day 0 to 160, for values of 1.55 kg, 1.20 kg, and 1.64 kg after conversion into the corrosion amount.

The currents of PHC-1 and PHC-2 displayed the same trend in the early stage, and hazardous ions did not penetrate the interior completely at this stage. After 22 d, the electric current of PHC-1 was larger than that of PHC-2. Due to the protective layer, hazardous ions had difficulty entering PHC-2. The final corrosion amount of PHC-2 was 0.35 kg, which was smaller than that of PHC-1. The current of PHC-3 in the drying and watering cycle environment tended to level off on day 75 and decreased again after 140 d. The corrosion amount of PHC-3 was higher than that of PHC-1, but only by 0.09 kg. This indicated that although the drying–wetting cycle can accelerate precast pile corrosion, this effect was smaller compared with the effect of electrification.

When the experiment was over, PHC-2 with the epoxy resin avoided 22.6% of the amount of corrosion compared to PHC-1, while PHC-4 in a drying–wetting cycle leachate environment increased the amount of corrosion by 5.8%.

**Figure 9:**
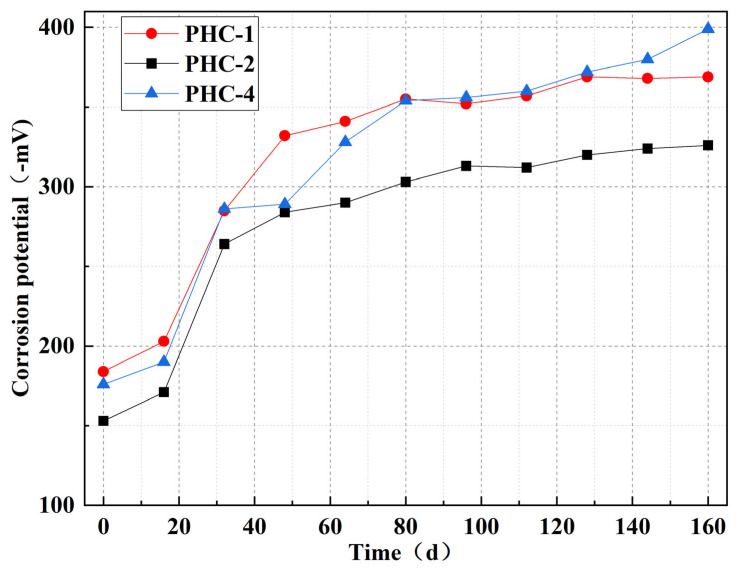
Corrosion potential changes in prestressed piles.

The corrosion potential of the test pile was measured every 16 d after energization, and energization was stopped 1 h before the test. The test piles were taken out, and the surface rust was cleaned. The grid area was divided according to the requirements. A steel bar was cut out and tested using a steel bar corrosion meter. Figure 9 illustrates the corrosion potential of the prestressed piles.

The change rule of the corrosion potential was similar to that of the current. Specifically, the three piles exhibited the same trend from day 0 to 32 but diverged from day 32 to 64. The corrosion potential of PHC-1 decreased faster in this stage and reached −341 mV on day 64. The corrosion potential of PHC-4 decreased rapidly after 48 d, approaching that of PHC-1 on day 80. The corrosion potential of these three piles tended to level off after 90 d, consistent with the changing trend of the electric current. On day 48, the corrosion potential of PHC-2 was −284 mV, 48 mV higher than that of PHC-1 and 5 mV higher than that of PHC-4. With the increase in time, there was a growing gap between PHC-2 and PHC-1 or PHC-4.

When the corrosion potentials of PHC-1, PHC-2, and PHC-3 were −369 mV, −326 mV, and −399 mV at the end of the experiment, there was an increase of 43 mV in the corrosion potential of PHC-2, which used the epoxy resin, and a decrease of 30 mV in the corrosion potential of PHC-4, which was subjected to the drying–wetting cycle.

The corrosion potential values of PHC-1 and PHC-4 were less than −350 mV on day 160, while those of PHC-2 coated with the epoxy resin protective layer were −324 mV and −326 mV on days 144 and 160, larger than −350 mV. According to the evaluation standard of the corrosion potential of steel reinforcement in structural concrete, PHC-1 and PHC-4 were at level 3 and PHC-2 was at level 2 [24].

### 3.3. Changes in Weld Depths

PHC piles are mainly connected by end plate welding to meet the design pile length requirements, and the durability of the welding in harsh environments needs to be emphasized.

**Figure 10:**
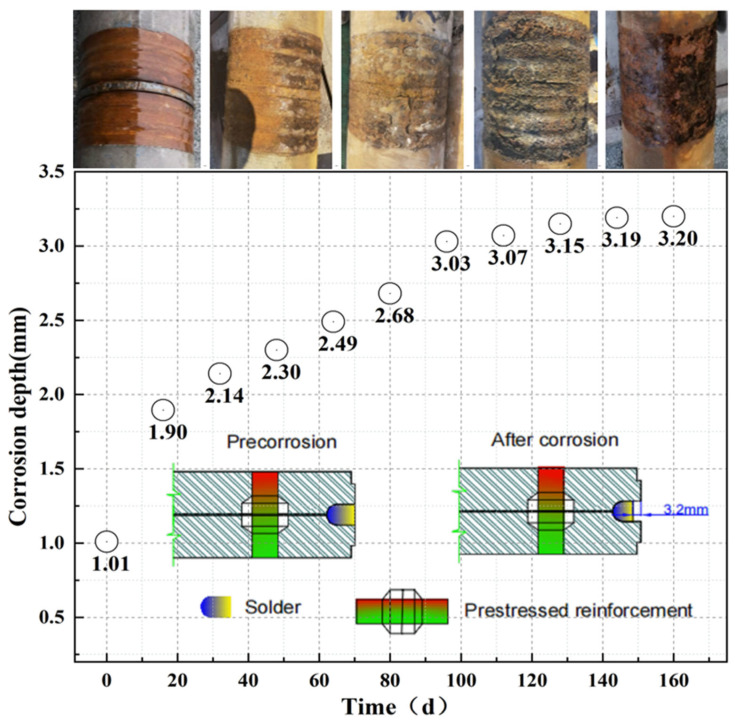
Changes in the corrosion depth of the welding seams.

The appearance of the welding seams of PHC-3 in harsh environments from day 0 to 160 is illustrated in Figure 10. The welding seam was a new one, with obvious metallic luster on day 0, and it was corroded by electricity by day 16. There was no metallic luster at the welding seam, and there were more rust deposits on the skirt plate and the welding seam. The thickness of the skirt plate was about 1.5 mm, and it had corroded completely within 50 years.

The depth change in the weld is illustrated in Figure 10. A large depth change was observed in the early stage and tended to level off in the later stage. The final corrosion depth was 3.2 mm.

The prestressed piles had a load value of 106 kN under the ultimate bending moment condition. When calculating the tensile bearing capacity of a single pile according to the connection strength of the prestressed concrete piles, the welded connection should be calculated according to the following formula:

(4) $$N\leq \frac{1}{4}\left(d_{1}^{2}-d_{2}^{2}\right)f^{w}$$

where *N* represents the single pile design value of the pullout force (kN); *d*_1_ represents the weld outer diameter (mm), 398 mm; *d*_2_ represents the weld inner diameter (mm), 376 mm; and *f^w^* represents the weld tensile strength design value, 170 MPa.

When the weld depth decreased, the weld outer diameter *d*_1_ and its pull-out bearing capacity decreased.

Table 3 shows the results corresponding to the corrosion allowance of the weld depth of pile joints under the simulated 50-year corrosive environment. This corresponds to the requirements of the pile cracking moment, and there was a considerable corrosion allowance for the welding seam. The accelerated test in the underground corrosive solution of the landfill indicated that the welded joints of prestressed piles had favorable durability after 50 years in the high Cl^−^ environment.

### 3.4. Discussion

The external current method can be employed to accelerate the diffusion of chloride ions in pile concrete; in this case, it energized and accelerated the prestressed piles for 160 d, equivalent to 50 years of service in the landfill landscape. In general, the steel corrosion rate in the prestressed piles decelerated with an increase in age. This may be attributed to a decrease in the cross-sectional area of the steel during corrosion, which increasingly blocked the passage of the electric current. Therefore, the corrosion rate of the piles reached the maximum within 16–32 d, followed by a gradual decrease in the corrosion rate of the subsequent prestressed piles.

When the prestressed piles were in an immersion environment, the chloride ion concentration in the surface layer of the pile concrete started to rise first. The concentration of the chloride ions inside the concrete began to rise in the middle and late stages until the concentration of the chloride ions inside the concrete was equal to that on the surface. This conformed to the permeation law of chloride ions in concrete. From day 0 to 16, the chloride ion concentration of the prestressed piles in the drying–wetting cycle environment was smaller than that of the prestressed pile concrete in the immersion environment. As the drying–wetting cycle and energization exerted more significant effects on chloride ion diffusion, the chloride ion concentration of PHC-4 was higher than that of PHC-1. The surface layer of the core sample of PHC-1 and PHC-4 can be stained white by AgNO_3_ on day 160. The drying–wetting cycle increased chloride penetration by 5.8%; a coating on the surface of pipe piles can effectively prevent the diffusion of chloride ions into the inside of the pipe piles, and the application of the epoxy resin reduced chloride penetration by 22.6%. This result was verified in the tests on electric current and half-cell corrosion potential, resulting in minimal rusting and maximum half-cell corrosion potential.

After the accelerated corrosion continued for 160 d, the skirt plate of the welded pile was corroded completely. The average change in the weld depth was 3.2 mm. Compared with the maximum corrosion depth of 6.53 m allowed by the standard, there was a large rust allowance to meet the requirements of the project.

The size, prestress, and reinforcement of prestressed piles will affect the corrosion. This paper only takes PHC 400(95) as the research object, which is the most used in a leachate environment, aiming to provide practical guidance for most projects.

The current acceleration experiment only accelerated the corrosion of chloride ions, and has little effect on sulfate and ammonia roots. How to couple the effects of these will be an interesting discussion point.

## 4. Conclusions

In the corrosion test of prestressed piles in a landfill leachate environment, the electrification-accelerated corrosion of prestressed piles has presented favorable durability. The implications of these findings for the design and maintenance of prestressed concrete structures in leachate environments are significant. In general, prestressed piles can meet the conditions of use in leachate-soaked environments with a building design life of 50 years. When in a drying–wetting cycle leachate environment, prestressed piles can also be used, but some protective measures are recommended. The specific conclusions are elucidated as follows:

(1) The chloride ion concentration in piles increased gradually with an increase in time and leveled off at the later stage of tests. The chloride ion concentration in the surface layer of the pile concrete began to rise first, while the internal chloride ion concentration started to rise in the middle and later stages. Finally, the internal chloride ion concentration was equal to the surface one.
(2) The corrosion rate of the pile in the drying–wetting cycle environment accelerated in the middle and late stages, and the drying–wetting cycle increased chloride penetration by 5.8% at 160 d. After 64 d, the corrosion potential of the pipe pile in the drying–wetting cycle decreased faster, and its resistance also began to increase faster. In addition, the current decreased faster, the chloride ion concentration increased rapidly, and the corrosion potential decreased faster.
(3) Applying a layer of epoxy resin protective layer on the pile can inhibit the chloride ion erosion and prevent the chloride ions from entering the pile concrete effectively, and the application of the epoxy resin reduced chloride penetration by 22.6% at 160 d. The chloride ion concentration was the lowest and the corrosion potential was the highest in the test piles coated with epoxy resin. In practical engineering applications, the test results showed that applying a protective layer on the surface of prestressed piles can improve the durability of precast piles.
(4) Under the accelerated corrosion for 160 d, the actual corrosion depth of the welding seam was 3.20 mm. Compared with the maximum corrosion depth of 6.53 mm permitted by the standard, there was a large corrosion allowance. Therefore, the prestressed piles can be welded in a leachate environment.

## Figures and Tables

**Table 1:** Chemical composition of cement and slag powder (%).

Chemical Composition	SiO_2_	Al_2_O_3_	Fe_2_O_3_	CaO	MgO	SO_3_	R_2_O	L.O.I.
Cement	19.40	4.73	2.98	64.04	2.30	2.58	0.68	2.80
Slag Powder	38.27	8.41	1.58	42.65	7.40	0.14	0.45	1.10

**Table 2:** The mix ratio of concrete (kg/m^3^).

Grade	Cement	Water	Fine Aggregate	Coarse Aggregate (5–16 mm)	Coarse Aggregate (16–32 mm)	Slag Powder	Water Reducer
C80	414	160	690	537	537	62	5.7

**Table 3:** The depth corrosion allowance of the welded joints of the prestressed piles under the simulated 50-year corrosive environment.

The Load Condition Corresponds to the Calculated Values of the Weld Depth (mm)			50-Year Equivalent Corrosion Depth (mm)	Corrosion Allowance (mm)
Loading Condition	Loading Intensity (kN)	Corresponding Weld Depth (mm)		
Cracking moment (kN·m)	64	4.36	3.20	4.44
Ultimate bending moment (kN·m)	106	6.53		2.27

## Data Availability

The raw data supporting the conclusions of this article will be made available by the authors on request.
